# Calibration of transition probabilities to model survival of adjuvant trastuzumab for early breast cancer in Indonesia

**DOI:** 10.1017/S0266462325000157

**Published:** 2025-03-26

**Authors:** Arie Rahadi, Rizki Tsalatshita Khair Mahardya, Putri Listiani, Eva Herlinawaty, Ryan Rachmad Nugraha, Dani Ramdhani Budiman, Christian Suharlim

**Affiliations:** 1 Management Sciences for Health, Arlington, VA, USA; 2 Center for Health Financing Policy and Insurance Management, Gadjah Mada University, Sleman, Yogyakarta, Indonesia; 3 Center for Health Financing and Decentralization Policy, Ministry of Health Republic of Indonesia, Central Jakarta, Jakarta, Indonesia

**Keywords:** calibrations, models, economic, survival, breast neoplasms, cost-effectiveness analysis

## Abstract

**Objectives:**

Cost-effectiveness models fully informed by real-world epidemiological parameters yield the best results, but they are costly to obtain. Model calibration using real-world data/evidence (RWD/E) on routine health indicators can provide an alternative to improve the validity and acceptability of the results. We calibrated the transition probabilities of the reference chemotherapy treatment using RWE on patient overall survival (OS) to model the survival benefit of adjuvant trastuzumab in Indonesia.

**Methods:**

A Markov model comprising four health states was initially parameterized using the reference-treatment transition probabilities, obtained from published international evidence. We then calibrated these probabilities, targeting a 2-year OS of 86.11 percent from the RWE sourced from hospital registries. We compared projected OS duration and life-years gained (LYG) before and after calibration for the Nelder–Mead, Bound Optimization BY Quadratic Approximation, and generalized reduced gradient (GRG) nonlinear optimization methods.

**Results:**

The pre-calibrated transition probabilities overestimated the 2-year OS (92.25 percent). GRG nonlinear performed best and had the smallest difference with the RWD/E OS. After calibration, the projected OS duration was significantly lower than their pre-calibrated estimates across all optimization methods for both standard chemotherapy (~7.50 vs. 11.00 years) and adjuvant trastuzumab (~9.50 vs. 12.94 years). LYG measures were, however, similar (~2 years) for the pre-calibrated and calibrated models.

**Conclusions:**

RWD/E calibration resulted in realistically lower survival estimates. Despite the little difference in LYG, calibration is useful to adapt external evidence commonly used to derive transition probabilities to the policy context, thereby enhancing the validity and acceptability of the modeling results.

## Introduction

Breast cancer is the most common malignancy in Indonesia, with an estimated annual incidence and mortality of 44.0 and 15.3 per 100,000, respectively (1). Reports suggest that 40–70 percent of women with breast cancer in Indonesia had late clinical presentation and diagnosis (2;3), or approximately three times as high in proportions compared to high-income countries (4). Claims of National Health Insurance (NHI) for cancer rank the second after cardiovascular disease in the catastrophic illness category (5), and these costs are projected to grow as the country is in the cusp of an epidemiological transition in health burden to noncommunicable diseases (6;7).

Trastuzumab is a recombinant, humanized, monoclonal antibody with a demonstrated efficacy as an adjuvant treatment to standard chemotherapy for early breast cancer, with durable treatment effects observed for up to 10 years (8–10). The treatment suppresses abnormal amplification of the human epidermal growth factor receptor 2 (HER2) gene or overexpression of its protein, which is linked to faster disease progression, higher rates of relapse, and mortality (11). The landmark joint analysis of the North Central Cancer Treatment Group and the National Surgical Adjuvant Breast and Bowel Project trials demonstrated significant improvements in disease-free survival (DFS) and overall survival (OS), with over one-third reduction in disease progression (hazard ratio (HR) = 0.63; 95 percent confidence interval (CI) = 0.54, 0.73; *p* < 0.001) and mortality (HR = 0.60; CI = 0.53, 0.68; *p* < 0.001) among patients who received 12-month adjuvant trastuzumab as compared to who received chemotherapy alone (9). An effect size of similar magnitude and durability has been confirmed in a subsequent study (10).

Despite this favorable efficacy profile, the high cost of trastuzumab, at an estimated $20,000 in global average for a 12-month treatment course (12), is unaffordable for many health systems and is likely to exceed the eligible ceiling for public health funding to be included in the NHI coverage (13). Published economic evaluations in low- and middle-income countries (LMICs) report that 12-month adjuvant trastuzumab was cost-effective in China (14) or Iran (15), but not in the Philippines (16) or Latin American countries (17;18) by standard cost-effectiveness eligibility of being not in excess of three times per capita gross domestic product in its incremental cost per health gain. A previous economic evaluation of trastuzumab for metastatic breast cancer in Indonesia reported an incremental cost-effectiveness ratio (ICER) of USD 17,307 per gain in quality-adjusted life-years (19). Although this figure exceeded three times per capita gross domestic product, trastuzumab for this indication received approval under NHI coverage in 2023 (20).

One critical input to a cost-effectiveness evaluation for health technology assessment (HTA) is a set of transition probabilities. In state-transition models that rely on a Markov process, a synthetic cohort moves (or “transitions”) from one health state to another with each passage of time (“cycle”) according to pre-defined probabilities until the entire cohort dies (the ultimate health state), from which no further transition is possible, or until the total number of cycles in units of time is exhausted (21). In a comparison of two (or more) treatment groups, transition probabilities in the reference, standard of care (SoC) group, are of paramount importance from which the transition probabilities for the other group(s) are calculated by multiplication with the corresponding relative treatment effects in HR or risk ratios. Real-world evidence (RWE), obtained from multiyear longitudinal real-world data (RWD) on local cohorts for whom coverage or pricing decisions are to be made, is best positioned to inform transition probabilities for its high contextualization with the policy decision and its representation of typical consumers of health technologies in the real-world setting (22;23). In many settings, such RWE may be unavailable or only partially available, for example, in shorter follow-up or incomplete (missing) observations, or for only a few segments of the patient population (24); and utilizing external evidence from multiple studies is a practical recourse to populate various transition probabilities and other model parameters.

In this study, we apply model calibration as a way to contextualize transition probabilities from external evidence for our Markov model of adjuvant trastuzumab. Model calibration compares the model projections with a target quantity from the observed RWE and iteratively calculates a new set of values for transition probabilities within their plausible range to provide the best fit to the target (25). The best model fit seeks to minimize the difference between the projected outcomes and those observed in RWE at a specified time point (e.g., 5-year survival), to which the initial transition probabilities are calibrated and the Markov model is adjusted to the new (calibrated) values (25). We feel that model calibration presents a valuable avenue to advance RWD/E utilization in HTA for its intuition, availability in standard decision-modeling packages, and non-exhaustive data requirements, all of which are features that appeal to jurisdictions with significant challenges in health data infrastructure, including ours.

The objective of the current study was therefore to: (a) calibrate the transition probabilities of a Markov model of adjuvant trastuzumab versus standard chemotherapy in HER2-positive, early breast cancer and (b) compare the survival outcomes on pre-calibrated and calibrated transition probabilities. The results of our model calibration will provide adjustments to the transition probability parameters for use in a cost-effectiveness evaluation of adjuvant trastuzumab for early breast cancer in Indonesia.

## Methods

This study provides a groundwork for a cost-effectiveness analysis of adjuvant trastuzumab, which at the time of writing was recruiting participants for data collection on patient utilities (EQ-5D-5L) and out-of-pocket outlays. The SoC treatment comprises approved chemotherapies in the oncologic practice of Indonesia, typically given every 21 days for up to eight cycles. Trastuzumab 8 mg/kg loading dose and 6 mg/kg every 21 days for 18 cycles is administered after the first four cycles of chemotherapy are completed for a total treatment duration of 52 weeks.

## Model structure

**Figure 1.**
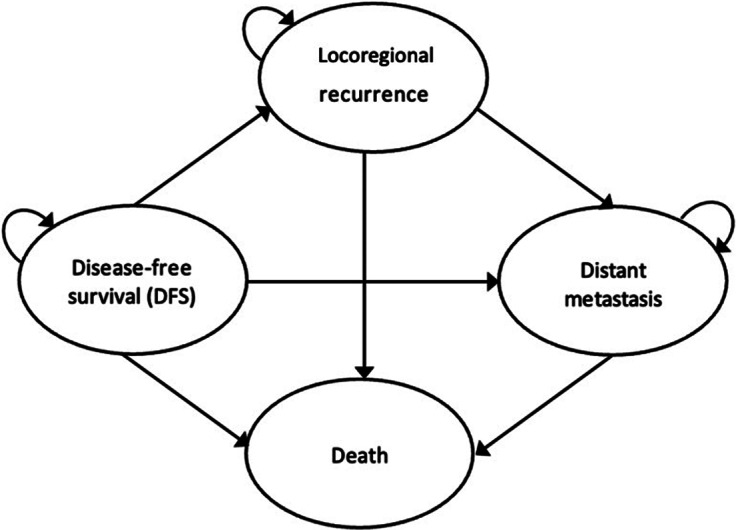
Model structure.

We developed a Markov cohort model comprising four health states: DFS, Locoregional Recurrence, Distant Metastasis, and Death for SoC and adjuvant trastuzumab (Figure 1). The cohort assumed a representative female patient aged 50 years with HER2-positive, early stage breast cancer (stages IA–IIIA) who initiated treatment and were disease-free at the start of analysis. After transitioning from DFS, a return to a less severe health state was not possible. The model was evaluated over a 50-year time horizon in annual cycles. Markov models were built using TreeAge Pro 2023 (Williamstown, MA) and Microsoft Excel®.

## Transition probabilities and treatment effects

The initial transition probabilities in the SoC group were informed by literature (9;10;18;19), selected to represent a plausible range and likely values (Table 1). Because 20 percent of the SoC participants in the source trial received adjuvant trastuzumab after completion of their primary chemotherapies (9), we applied adjustments in health state transitions from DFS within the SoC group using the rate difference by crossover status from another trial (10). The mortality risk of cardiotoxicity was built into the transition probability from DFS to death for each treatment group from a 10-year cumulative incidence (1.1 percent and 5.4 percent for the SoC and trastuzumab groups, respectively) from the same trial (10).

**Table 1.** Initial transition probabilities of the chemotherapy group and treatment effects of adjuvant trastuzumab

Parameter	Mean (Plausible range)	Distribution	Remarks, source
1. Transition probabilities			
DFS to recurrence	0.0098 (0.0038; 0.0217)	Beta	Perez (9) with adjustment for treatment crossover from Cameron (10).
DFS to metastasis	0.0267 (0.0142; 0.0525)	Beta	Perez (9) with adjustment for treatment crossover from Cameron (10).
DFS to death	0.0358 (0.0199; 0.0667)	Beta	Perez (9) with adjustments for additional mortality from congestive heart failure and treatment crossover from Cameron (10).
Recurrence to metastasis	0.0777 (0.0521; 0.1198)	Beta	Botelho (18)
Recurrence to death	0.0794 (0.0414; 0.1554)	Beta	Allen (26)
Metastasis to death	0.2905 (0.1811; 0.4137)	Beta	Kristin (19)
2. Treatment effects of trastuzumab			
Pooled HR for DFS		Log-normal	Estimated from meta-analysis^a^
Up to year 5	0.43 (0.34; 0.54)		
Year 6	0.47 (0.39; 0.57)		
Year 7	0.52 (0.45; 0.60)		
Year 8	0.57 (0.52; 0.63)		
Year 9	0.63 (0.59; 0.67)		
Year 10	0.69 (0.65; 0.73)		
Year 11	0.76 (0.70; 0.82)		
Pooled HR for OS			
Overall	0.67 (0.57; 0.79)	Log-normal	Estimated from meta-analysis^a^

DFS: disease-free survival; HR: hazard ratio; OS: overall survival.

a See Supplementary Material 1 for details on varying treatment effects by follow-up duration for DFS.

We pooled the treatment effects of adjuvant trastuzumab for DFS (HR_pooled_ = 0.67; CI = 0.52, 0.87; *P* = 0.016; I^2^ = 0.633) and OS (HR_pooled_ = 0.67; CI = 0.57, 0.79; *P* = 0.004; I^2^ = 0.203) from four major trials (9;10;27;28). These pooled HRs were applied to the transition probabilities of SoC for health state transitions from DFS to obtain the corresponding transition probabilities of the trastuzumab treatment group. Therefore, adjuvant trastuzumab was assumed to have no effect on prognosis once the cancer recurred locoregionally or distantly. Because the magnitude of HRs for DFS varied by trial follow-up duration, we applied time-varying treatment effects specific to the first five cycles (years), corresponding to the minimum follow-up duration in the trials, and to each subsequent cycle until 11 years. We assumed that treatment effects would last for 11 years as per the longest follow-up during which between-group differences persisted (10). From year 12 onward, mortality and all other transitions from DFS for adjuvant trastuzumab referenced the SoC rates, as defined by a time-exponential function of the baseline transition probabilities. Supplementary Material 1 provides information on study eligibility and the meta-analysis methodology used to pool study outcomes.

### Outcome measures

We computed OS duration, measured in the number of years spent in health states other than death, and the life-years gained (LYG) from adjuvant trastuzumab, defined as the difference in OS duration between the treatment groups. Outcomes were projected over the analysis time horizon of 50 years.

## RWE of OS

We abstracted data from cancer registries at two major hospitals in Jakarta and Yogyakarta on 1,007 female patients (51 years in average age) diagnosed with stages IA–IIIA breast cancer during 2016–2019, who received chemotherapy without trastuzumab, with follow-up through December 2022 (median: 24 months). The Jakarta hospital is a national-referent hospital for cancer care and has the largest volume of cancer patients in the country. The Yogyakarta hospital is a regional-referent hospital for cancer care. The ethics committees of Gadjah Mada University and the respective hospitals approved the use of the registry data for the study. One hundred and twenty-two patients died (12 percent) over 84 months, with most deaths occurring within the first 24 months (n = 97), including half (n = 60) during the second year alone. Over 60 percent of the cohort were censored at the last visit date and no longer contributed survival data beyond 36 months, thereby likely misrepresenting the low number of deaths in the later period of the registry. Given this limitation, we computed RWE survival in the chemotherapy group at 2 years of follow-up, which was 86.11 percent (CI = 83.21, 88.54). This time point corresponds to the average follow-up period and falls within the period of peak mortality (29;30). We set this survival proportion as the target OS to calibrate the initial values of transition probabilities for the SoC group. Supplementary Material 2 shows the Kaplan–Meier curve of RWE OS.

## Calibration techniques

We compared the performances of three optimization methods in calibration: (a) Nelder–Mead simplex; (b) Bound Optimization BY Quadratic Approximation (BOBYQA); and (c) generalized reduced gradient (GRG nonlinear). These methods were chosen because of their availability in standard modeling packages and ease of implementation. The Nelder–Mead algorithm is a direct search method that iteratively transforms the simplex using the plausible range of transition probabilities to find the optimal solution that minimizes the discrepancy between the projected 2-year OS and its observed RWE rates (31). Similar to the Nelder–Mead method, BOBYQA employs a derivative-free algorithm. In finding an optimal solution, BOBYQA utilizes a trust region procedure to iteratively update the values of the transition probabilities around neighboring values within the region using quadratic approximations (32). By contrast, the GRG nonlinear method uses partial derivatives in combination with nonlinear programming to find the optimal solution (33). Of the three optimization methods, BOBYQA and GRG nonlinear are less sensitive to local minima than Nelder–Mead (32).

## Data analysis

We implemented the Nelder–Mead and BOBYQA optimization in TreeAge Pro 2023 using the calibration menu and set the optimization thresholds to 1E-10 and 1E-13 in relative and absolute terms, respectively. The GRG nonlinear was implemented using the Solver add-in in Microsoft Excel®, with an equivalent relative threshold and 200 sets of starting values of transition probabilities within their plausible bounds. Satisfactory model fit was achieved if the projected 2-year OS falls within the CI coverage of the RWE OS (25). The goodness of fit was judged by the difference in the 2-year OS that each optimization method projected relative to the RWE, with a smaller value indicating a better fit to the RWE OS. Model projections of OS duration and LYG with parameterization using the pre-calibrated and calibrated transition probabilities were compared. Bias-corrected CIs (CI_bc_) and *P* values (*P*_bc_) were calculated from a beta and log-normal distribution of 10,000 Monte Carlo draws for transition probabilities and treatment effects, respectively. All outcomes were discounted at a 3 percent rate per year.

## Results

## Calibration performance and results

The projected 2-year OS was 92.25 percent in contrast to the observed RWE at 86.11 percent. All the optimization methods produced a satisfactory fit. However, the GRG nonlinear had the best goodness of fit with the smallest difference (1.64E-14) compared to the Nelder–Mead (1.89E-07) or BOBYQA optimization (6.18E-11) (Table 2). The calibrated transition probabilities from DFS to death significantly increased from the initial, pre-calibrated values, approaching the plausible upper bound for all three optimization methods. Calibration also significantly increased the transition probabilities leading to metastasis either from DFS or from recurrence. All methods targeted these transitions because of their role in terminating cohort membership from death or elevating its risk in the metastasis health state.

**Table 2.** Calibrated transition probabilities of the chemotherapy group

Transition probabilities	Initial, pre-calibrated	Calibrated values					
		Nelder–Mead		BOBYQA		GRG nonlinear	
	values (SE)	Mean (SE)	% change	Mean (SE)	% change	Mean (SE)	% change
DFS to recurrence	0.0098	0.0217*	121%	0.0125	28%	0.0118	20%
	(0.0023)	(0.0051)		(0.0029)		(0.0028)	
DFS to metastasis	0.0267	0.0501*	88%	0.0524*	97%	0.0350	31%
	(0.0044)	(0.0083)		(0.0087)		(0.0058)	
DFS to death	0.0358	0.0651*	82%	0.0655*	83%	0.0667**	87%
	(0.0055)	(0.0100)		(0.0101)		(0.0103)	
Recurrence to metastasis	0.0777	0.1071*	38%	0.0782	1%	0.1182**	52%
	(0.0084)	(0.0116)		(0.0084)		(0.0128)	
Recurrence to death	0.0794	0.1041	31%	0.0796	0%	0.1008	27%
	(0.0135)	(0.0176)		(0.0135)		(0.0171)	
Metastasis to death	0.2905	0.3077	6%	0.2932	1%	0.3389	17%
	(0.0596)	(0.0631)		(0.0602)		(0.0695)	
Diff. with target 2-year OS	-	1.89E–07		6.18E–11		1.64E–14	

BOBYQA: Bound Optimization BY Quadratic Approximation; DFS: disease-free survival; GRG: generalized reduced gradient; OS: overall survival; SE: standard error. Stars denote a statistically significant difference between calibrated and pre-calibrated values at *P*_bc_ < 0.050 (*) or *P*_bc_ < 0.010 (**), computed from normally distributed Monte Carlo draws with the expected mean difference and combined variance.

By magnitude, when compared to the other optimization methods, the calibrated transition probabilities in the DFS health state under the GRG nonlinear were higher for transitions to death (+87 percent change from initial values) and metastasis (+52 percent change), but lower for transitions to recurrence (+20 percent change) (Table 2). In finding the optimal solution, the Nelder–Mead and BOBYQA, respectively, maximized the values of transition probabilities for transitions from DFS to recurrence and metastasis, whereas the GRG nonlinear leveraged transitions from DFS to death and from recurrence to metastasis. On average, changes in the remaining transition probabilities after calibration were appreciable (from recurrence to death) or minor (from metastasis to death) for all methods.

## Comparison of projected OS and LYG

**Figure 2.**
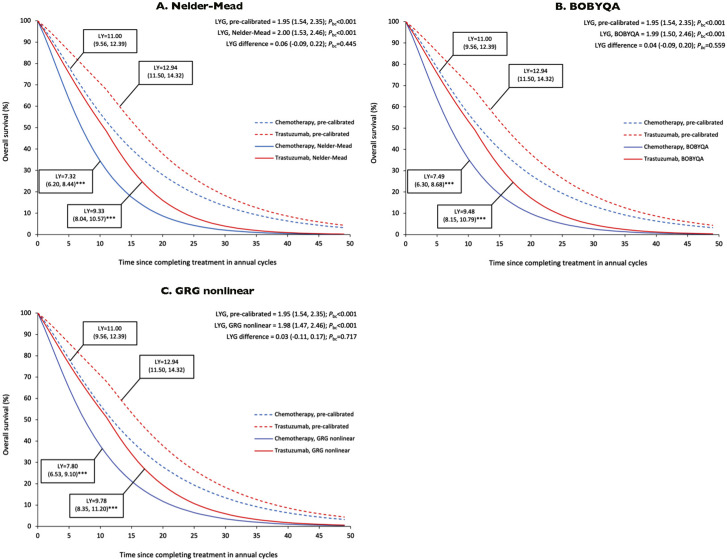
Projected overall survival and life-years gained. BOBYQA: Bound Optimization BY Quadratic Approximation; GRG: generalized reduced gradient; LY: life-years; LYG: life-years gained. Stars denote the statistical comparison of life-years with pre-calibrated values within a treatment group (*P_bc_ < 0.050; **P_bc_ < 0.010; ***P_bc_ < 0.001). All statistical tests and the 95% confidence intervals were based on the Monte Carlo empirical distribution with bias corrections.

The pre-calibrated OS duration was 11.00 years (CI_bc_ = 9.56, 12.39) for SoC and 12.94 years (CI_bc_ = 11.50, 14.32) for adjuvant trastuzumab (Figure 2). The calibrated OS duration averaged ~7.50 years for SoC and ~9.50 years for adjuvant trastuzumab across all optimization methods. All calibrated estimates were significantly shorter in both treatment groups (*P*_bc_ < 0.001), suggesting an overestimation of the pre-calibrated survival projections. The calibrated survival curves display a steeper downward slope that more realistically corresponds to the average life expectancy of the female population.

Adjuvant trastuzumab prolonged OS duration by approximately 2 years in the pre-calibrated model (LYG = 1.94; CI_bc_ = 1.54, 2.35; *P*_bc_ < 0.001) and calibrated model for all optimization methods (LYG = ~2.00; *P*_bc_ < 0.001) (Figure 2). The difference with the pre-calibrated LYG was small and statistically non-significant for the Nelder–Mead (∆LYG = 0.06; CI_bc_ = −0.09, 0.22; *P*_bc_ = 0.445), BOBYQA (∆LYG = 0.04; CI_bc_ = −0.09, 0.20; *P*_bc_ = 0.559), and GRG nonlinear methods (∆LYG = 0.03; CI_bc_ = −0.11, 0.17; *P*_bc_ = 0.717).

## Discussion

Calibration was used in this study to adjust transition probabilities and model projections against RWE survival. Survival projections using pre-calibrated transition probabilities overestimated the mean survival by around half as much as the calibrated values for SoC (11.00 vs. ~7.50 years) and by more than one-third as much for adjuvant trastuzumab (12.94 vs. ~9.50 years). Calibration, as applied in this study, shifted the initial survival curve downward with a proportional change in survival rates. The survival benefits of LYG were similar between the pre-calibrated and calibrated models.

The GRG nonlinear method had the best fit, and the transition probabilities thus calibrated can be used to inform a cost-effectiveness analysis of adjuvant trastuzumab for HER2-positive early breast cancer in the country. In comparison with other studies, our calibrated estimates of OS duration for SoC (7.80 years) and trastuzumab (9.78 years) are lower than the estimates for China (14), Iran (15), and Thailand (34) where a wide range of projected OS duration for standard chemotherapy (8.40–15.30 years) and trastuzumab (15.82–18.20 years) was reported. Our calibrated estimates of LYG broadly fall in the high range, but were less than those reported for China (14) or Thailand (34). Interestingly, studies reporting both greater OS duration and LYG of adjuvant trastuzumab also assumed a shorter duration of treatment effects than in this study (14;34), which would have led to lower estimates in our model. This incongruity may be attributable to variations in input parameters, model structures, and other aspects of modeling that are specific for the decision context in the respective study jurisdictions. The RWE OS in this study mirrors the practice in service delivery where a shortfall in the supply of health human resources negatively impacts on access to and the quality of oncologic care (2;35;36). This health system context was thus captured and represented in the survival projections by virtue of model calibration.

Our study is the first known official HTA evaluation in the country to apply calibration to empirical RWE. We also compared different optimization methods, all of which passed the standard fit criteria in relation to the observed RWE and provided comparable survival projections. However, some key limitations of this study should be acknowledged. First, the limited scope of RWD encompassing two major hospitals may underrepresent patients with early breast cancer at the national level, rendering the results less generalizable to other settings of care providers, particularly those accessing care other than at high-caseload hospitals.

Second, we used only a single target, 2-year OS, in the calibration process. Both the follow-up duration and high attrition in the registry-based RWD are major limitations. The decision to focus on this early survival period in our calibration exercise was motivated by the need to anchor the model and its long-term calibrated projections to a time interval for which robust data were available and during which the risk of mortality is increasing (29;30). However, this means that the trajectory of OS projections would proceed at a slope reflecting these high early mortality rates in the remaining time horizon, potentially underestimating survival in both treatment groups, particularly if the true mortality rates rapidly declined at subsequently nearby time points. Additional time points covering a meaningful portion of the model’s time horizon would help reduce uncertainties in our calibrated projections. We anticipate that the uncertainties surrounding the calibration targets, currently undefined in this study, will have the largest impact on our cost-effectiveness findings if they substantively influence the projected survival gains (37).

Establishing registries that integrate and harmonize data across key care providers is a crucial step toward ensuring the quality generation of RWE for economic evaluations in the country. While this process is ongoing and in the absence of empirical data to inform uncertainties in long-term survival, structured expert elicitation, which seeks to formally estimate uncertain quantities from expert knowledge in a probability distribution (38), offers a valuable avenue. This method is frequently applied to extrapolate long-term treatment benefits or survival over periods of up to 20 years in published studies (39) and frames uncertainties in the knowledge of local experts, rather than relying on evidence from different country contexts. Additionally, future evaluations may attempt calibrating against more than one index of key transitional drivers and at different time points concurrently, as more quality RWD becomes available or by utilizing structured expert elicitation.

Finally, the full implications of our calibrated model for costs and cost-effectiveness remain to be explored, as data collection on costs and health utilities was still ongoing at the time of writing this manuscript. However, we surmise that while the downward corrections in LYG will lower cost estimates in both treatment groups, the change is likely to be less pronounced for the trastuzumab group compared to SoC due to its high drug cost, which could result in larger incremental costs and ICER than pre-calibrated estimates. For instance, by parameterizing the model with direct medical costs from a trastuzumab study in the Philippines (16), our calibrated model using GRG nonlinear optimization shows a borderline statistical difference, with a modest 8.22 percent increase in incremental costs (CI_bc_ = 3.57, 16.43; *P*_bc_ = 0.004) and a similar 6.56 percent increase in ICER per LYG (CI_bc_ = −0.43, 15.37; *P*_bc_ = 0.065). We anticipate corrections of similar magnitude to ICER in our upcoming cost-effectiveness analysis results.

Our study illustrates the realities of incorporating RWD/E for cost-effective assessments of health technologies within the constraints of the country’s fragmented health system (40). The regulatory framework for population-based cancer registries has been in place since 2016 involving 15 regional and national referral hospitals (20). However, its current unavailability suggests the need for greater investments in realizing this objective. Although our data acquisition was somewhat partial and ad hoc, it is preferable to forgoing HTA due to a perceived lack of viable RWE or capacity to conduct HTA, which are listed among the common reasons to avoid HTA (41).

The RWD/E application in HTA has gained traction globally and in Asia to strengthen the conduct of cost-effectiveness assessments to fill the evidence gap from randomized trials in a local setting or boost the applicability of such an assessment to the setting where coverage or pricing decisions are to be made (42). With regard to input parameters, the use of RWD/E is imperative to derive disease background parameters such as mortality or prevalence (43), essential to inform key parameters that cannot be generalized from external settings such as costs and health utilities (35;43), and strategic to validate experimental evidence in the practice setting (44;45). Transition probabilities of the reference treatment are a product of incidence rates of various health events in the natural course of a disease, which extended longitudinal observations are best to inform (46). However, acquiring such rich RWD to construct a full set of RWE-informed transition probabilities is infeasible or highly costly within the remit of the surveillance system of many LMICs (47). Model calibration can therefore be a pragmatic solution to compromise this gap between the high demand for quality, extensive RWD/E and its short supply.

In this view of pragmatism, model calibration works to align transition probabilities extracted from multiple studies, which may inadequately represent the practice setting of a new health technology, with the existing RWE at the local level. We believe that advocating the utilization of existing RWD/E with a fair assessment of its inherent bias should be pursued for better informed policy decisions. The annual number of HTA submissions processed by the Indonesia’s Health Technology Assessment Committee and the National Formulary can reach hundreds and is expected to grow steadily (Personal Communication). In this setting, RWE calibration can lend more credence to assessment results in a more accessible way by utilizing one or more RWE data points to target in model projections, which also enhances the face validity of the results to policy makers (25). Noticeable differences between the pre-calibrated and calibrated results should, at minimum, lead to a discussion for more informed decision making or be presented as a form of sensitivity analysis to enhance the credibility of the modeling results.

## Conclusions

Our calibrated transition probabilities produced survival projections that were more congruent with the observed RWE for the treatment of early breast cancer in Indonesia. The pre-calibrated transition probabilities overestimated OS duration, which could potentially confound the results when interacting with cost parameters in a cost-effectiveness analysis. Our model calibration had low data burden and provides an example of how model refinements for more credible decision making in HTA can be performed within the constraints of the existing health data infrastructure for RWE in LMICs. Model calibration can be applied with relative ease in other disease areas, and its applications can encourage the validation of model parameters and promote RWD/E utilization toward advancing the health data infrastructure.

## Supporting information

Rahadi et al. supplementary material Rahadi et al. supplementary material

## Data Availability

Summary data from cancer registries, calibration macros (.xlsm) and the TreeAge model (.trex) used in the analysis of the current study are available from the corresponding author upon reasonable request.

## Supplementary material

The supplementary material for this article can be found at http://doi.org/10.1017/S0266462325000157.
