# Supplementary material for: Calibration of transition probabilities to model survival of adjuvant trastuzumab for early breast cancer in Indonesia
Source: Int J Technol Assess Health Care. 2025 Mar 26;41(1):e18. doi: 10.1017/S0266462325000157 (PMC11955306; doi:10.1017/S0266462325000157)
Supplement: Rahadi et al. supplementary material [file S0266462325000157sup001.docx]

**CALIBRATION OF TRANSITION PROBABILITIES TO MODEL SURVIVAL OF ADJUVANT TRASTUZUMAB FOR EARLY BEAST CANCER IN INDONESIA**

Supplementary Material

**Table of contents**

Supplement 1: Meta-Analysis of Treatment Effects of Adjuvant Trastuzumab 2

Supplement 2: Kaplan-Meier Curve of Real-World Evidence Overall Survival 4

**Supplementary Material 1: Meta-analysis of Treatment Effects of Adjuvant Trastuzumab**

We retrieved publications using the keywords "adjuvant trastuzumab" or "herceptin" and "breast cancer" and "HER2-positive" with a language limiter (English). Two researchers independently screened the abstracts and titles of the retrieved records and excluded reports of neoadjuvant chemotherapy, observational studies, and chemotherapies not commonly used in Indonesia's oncologic practice. Four eligible studies were included (1-4) from which we extracted the hazard ratio (HR) and corresponding 95% confidence interval (CI) for disease-free survival (DFS) and overall survival (OS). Adjusted treatment effects were prioritized in extraction, and crude risk ratios were extracted to approximate the HR in one study (1). We performed random-effects meta-analyses using the restricted maximum likelihood estimator and the Knapp-Hartung adjustment to the standard error to account for greater uncertainty in the pooled effect size.

*Disease-free survival*

Adjuvant trastuzumab reduced the risk of cancer recurrence by 33% (HR_pooled_ = 0.67; CI=0.52-0.87; *P* = 0.016; I^2^ = 63.3%) (**Figure S1A**). There was no evidence of small-study effects (*P*_Egger_ = 0.318). In meta-regression analysis, follow-up duration positively and significantly moderated the HR of DFS in that there was an 0.8% increase in HR (ratio of HR = 1.008; CI = 1.004-1.012; *P* = 0.013; I^2^ = 0.0%) for every additional month of follow-up. We calculated HR predictions over the observed range of follow-up duration (62 to 132 months) and used these time-varying estimates to reference the benefit in DFS from up to approximately five years (HR_pooled_ = 0.43; CI = 0.34-0.54; *P* = 0.004), the minimum follow-up duration, to 11 years since treatment completion (HR_pooled_ = 0.76; CI = 0.70-0.82; *P* = 0.004), the maximum follow-up duration across the included studies, for our calibration and survival projections.

*Overall survival*

Adjuvant trastuzumab reduced the risk of death by 33% (HR_pooled_ = 0.67; CI = 0.57-0.79; *P* = 0.004; I^2^ = 20.3%) (**Figure S1B**). Similarly, the Egger's test showed no evidence of small-study effects (*P*_Egger_ = 0.420). Follow-up duration did not moderate the HR of OS in the meta-regression analysis (*P* = 0.136), and we used the overall pooled effect in modelling survival projections, constant throughout all model cycles.

**Figure S1.** Forest plot of treatment effects of adjuvant trastuzumab

| **A. Disease-free survival** |
| --- |
| 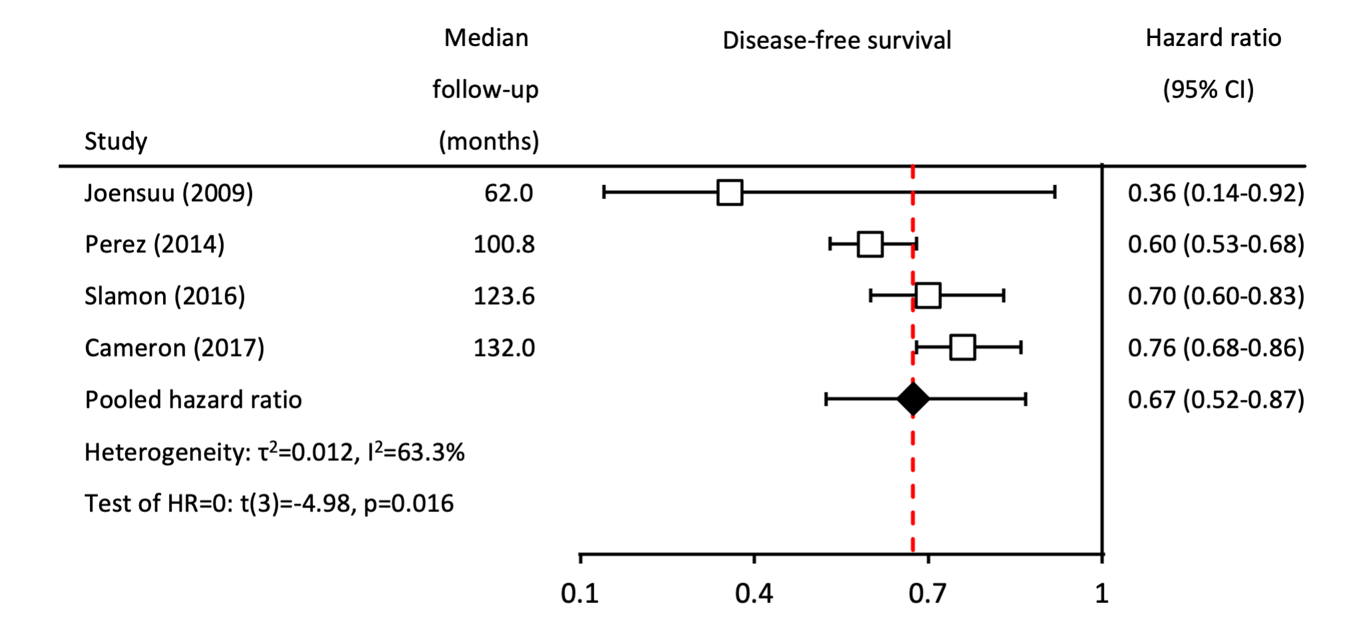 |
| **B. Overall survival** |
| 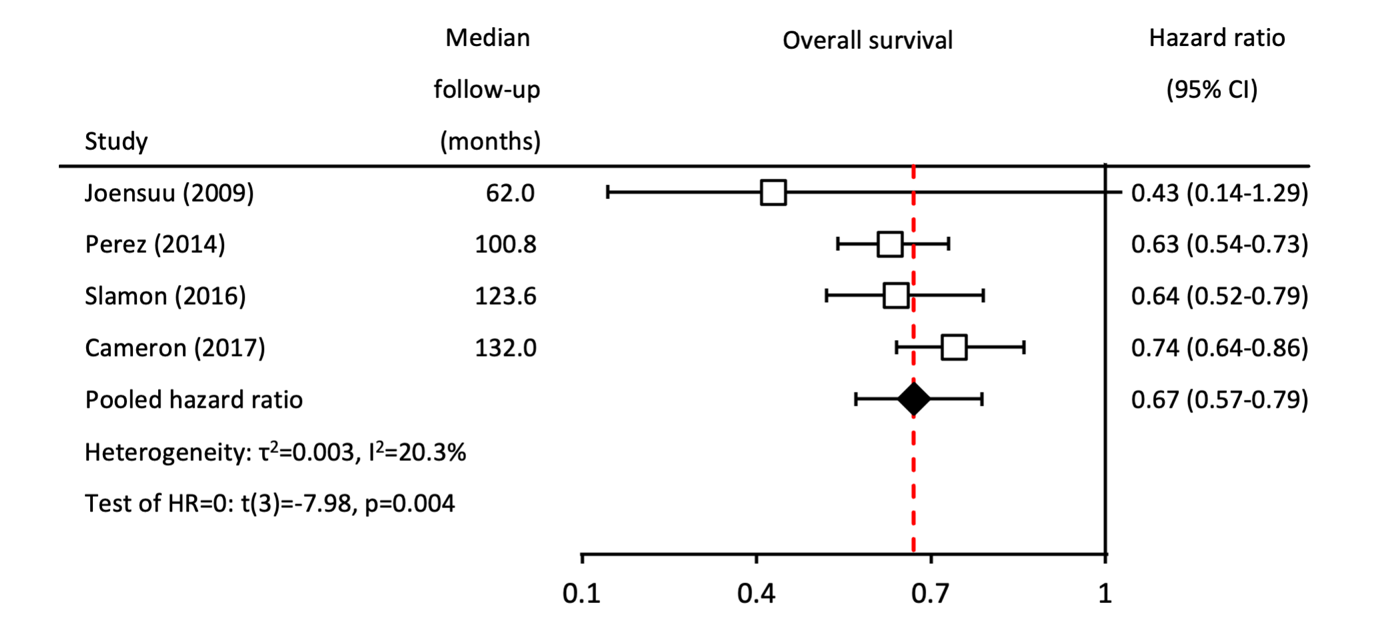 |
| CI: 95% confidence interval; HR: Hazard ratio.  **Chemotherapy regimens included:**   - Joensuu (2009): Docetaxel/fluorouracil, epirubicin, and cyclophosphamide (FEC). - Perez (2014): Doxorubicin and cyclophosphamide with or without paclitaxel. - Slamon (2016): Doxorubicin and cylclophosphamide. - Cameron (2017): Doxorubicin or epirubicin with cyclophosphamide and with or without 5-fluorouracil or methotrexate; doxorubicin or epirubicin and paclitaxel or docetaxel; cyclophosphamide and methetrexate and 5-fluorouracil. |

**Supplementary Material 2: Kaplan-Meier Curve of Real-World Evidence Overall Survival**

| 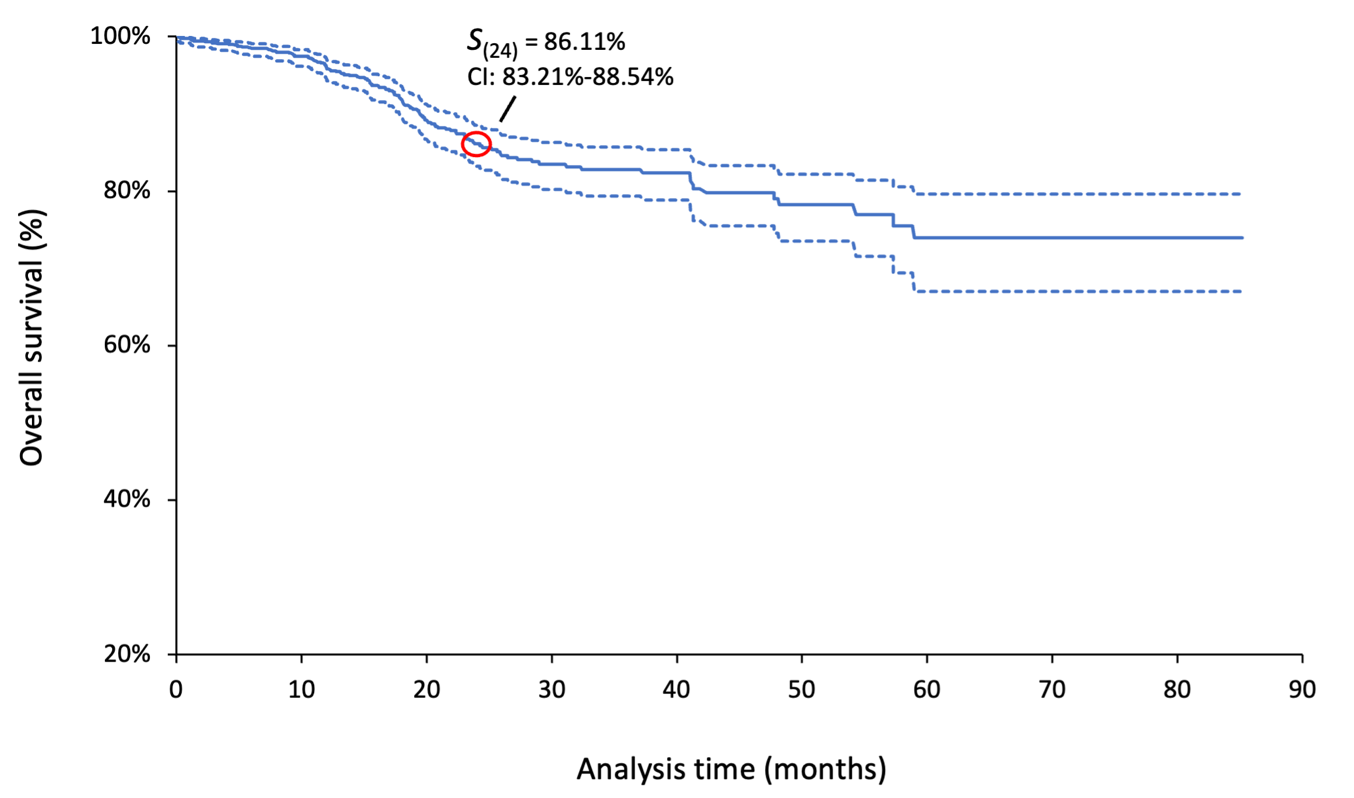 |
| --- |
| CI: 95% confidence interval  The graph shows the probabilities of overall survival (solid line) along with the 95% confidence interval (dashed line). |

**REFERENCES**

1. Joensuu H, Bono P, Kataja V, Alanko T, Kokko R, Asola R, et al. Fluorouracil, epirubicin, and cyclophosphamide with either docetaxel or vinorelbine, with or without trastuzumab, as adjuvant treatments of breast cancer: final results of the FinHer trial. J Clin Oncol. 2009;27(34):5685-92.

2. Perez EA, Romond EH, Suman VJ, Jeong JH, Sledge G, Geyer CE, Jr., et al. Trastuzumab plus adjuvant chemotherapy for human epidermal growth factor receptor 2-positive breast cancer: planned joint analysis of overall survival from NSABP B-31 and NCCTG N9831. J Clin Oncol. 2014;32(33):3744-52.

3. Slamon DJ, Eiermann W, Robert NJ, Giermek J, Martin M, Jasiowka M, et al. Ten year follow-up of BCIRG-006 comparing doxorubicin plus cyclophosphamide followed by docetaxel (AC→T) with doxorubicin plus cyclophosphamide followed by docetaxel and trastuzumab (AC→TH) with docetaxel, carboplatin and trastuzumab (TCH) in HER2+ early breast cancer. Cancer Research. 2016;76(Suppl 4):S5-04.

4. Cameron D, Piccart-Gebhart MJ, Gelber RD, Procter M, Goldhirsch A, de Azambuja E, et al. 11 years' follow-up of trastuzumab after adjuvant chemotherapy in HER2-positive early breast cancer: final analysis of the HERceptin Adjuvant (HERA) trial. Lancet. 2017;389(10075):1195-205.
